# Roles of Bile-Derived Exosomes in Hepatobiliary Disease

**DOI:** 10.1155/2021/8743409

**Published:** 2021-01-13

**Authors:** Yuan Yao, Dechao Jiao, Zhaonan Li, Xueliang Zhou, Jing Li, Zaoqu Liu, Xinwei Han

**Affiliations:** ^1^Department of Interventional Radiology, The First Affiliated Hospital of Zhengzhou University, Zhengzhou, 450052 Henan, China; ^2^Academy of Medical Sciences, Zhengzhou University, Zhengzhou, 450052 Henan, China

## Abstract

Exosomes are vesicles with a diameter of 30-150 nm produced by living cells and secreted into the extracellular matrix. Exosomes mediate cellular communication by carrying active molecules, such as nucleic acids, proteins, and liposomes. Although exosomes are found in various body fluids, little is known about bile-derived exosomes. This review is the first to summarize the methods of bile storage and isolation of biliary exosomes, highlighting the roles of bile-derived exosomes, especially exosomal noncoding RNAs, in physiological and disease states and discussing their potential clinical applications.

## 1. Background

Exosomes are vesicles secreted into the extracellular environment by the fusion of specific endosomes, i.e., multivesicular bodies (MVBs), with the plasma membrane; exosomes mediate cell-to-cell communication during many biological processes, including tumour progression and immune response [1, 2]. Exosomes are small (30–150 nm) vesicles with unique RNA and protein cargoes. The stability of noncoding RNAs in the exosomes is higher than that in body fluids; this increased stability may be due to the membranous structure of the outer vesicle surface. Exosomes are found in a wide variety of body fluids, including blood, urine, breast milk, amniotic fluid, saliva, cerebrospinal fluid, and ascites. Expansive distribution of exosomes has facilitated their extraction and research. Bile is a special body fluid that is secreted by the liver cells, stored in the gallbladder, and discharged into the duodenum through the bile duct to facilitate the digestion of fat and the absorption of fat-soluble vitamins. As a microenvironment for the growth of tumour cells, bile may contain a relatively high amount of tumour-related proteins and genes secreted by tumour cells and may thus be used as a source of early tumour markers and therapeutic targets for hepatobiliary diseases [3–5]. To date, exosomes in bile have been relatively rarely investigated, which may be due to the difficulty of bile collection and poorly developed methods of extraction of the exosomes from bile. This review summarizes feasible extraction methods and mechanisms and highlights potential clinical applications of biliary exosomes.

## 2. Overview of Exosomes

**Figure 1:**
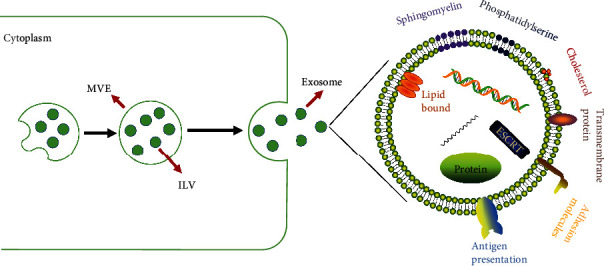
The structure, production, and secretion of the exosomes. Exosomes are membrane vesicles with a diameter of 30-110 nm produced by living cells. Exosomes are loaded with various types of antipresentation and adhesion molecules, transmembrane proteins, sphingomyelin, phosphatidylserine, etc. Exosomes carry a variety of active molecules, such as nucleic acids, which can mediate communications between the cells. Early exosomes in the cytoplasm are wrapped in MVEs in the form of ILVs, and exosomes are released from the cells after fusion of the vesicles with the outer membrane.

In the 1990s, Johnstone et al. identified a membranous vesicle in the supernatant of in vitro culture medium of sheep erythrocytes and named it an exosome [6, 7]. Exosomes are formed by intracellular lysosomal microparticles and released into the extracellular matrix after the fusion of the extracellular plasma membrane layer with the outer membrane of the vesicles. Exosomes are membranous vesicles secreted by living cells with a diameter of approximately 30-150 nm and a typical morphology. Exosomes contain mainly fusion proteins and transporters, heat shock proteins (e.g., HSP70), CD proteins, phospholipases, and other lipid-related proteins [8, 9]. These proteins constitute the structural basis for exosome identification (Western blotting). Moreover, exosomes from various cell sources have significant heterogeneity, and the cargo loaded in the exosomes varies depending on cell type and/or different states of the same cell [10]. Exosomes carry proteins, miRNAs, lncRNAs, circRNAs, and mRNAs involved in cell signal transduction and participate in the regulation of important activities and cell-to-cell communication [11, 12]. Furthermore, exosomes are widely distributed in almost all tissues, including interstitial tissues, and body fluids and play important roles in immune regulation, tumour metastasis, angiogenesis, and the initiation and development of disease [13, 14]. Given these characteristics, exosomes may be used as biomarkers for the early diagnosis and prognosis of cancer patients [15]. In addition, the membranous structure of the outer layer of the exosomes protects their internal cargo from clearance and degradation and provides sufficient stability that enables the use of the exosomes in drug delivery [16]. Thus, this newly defined treatment system may be used to increase the duration and efficacy of drug action (Figure 1).

## 3. Isolation and Characterization of Human Biliary Exosomes

### 3.1. Collection and Storage of Bile

Bile samples are obtained at ERCP or at the time of percutaneous manipulation of biliary tubes by interventional radiology (IR). Then, the samples are placed into a sterile collection tube. In previous studies, bile was always extracted without contrast injection to ensure exosome yield and to reduce the interference of irrelevant factors [17–19]. Cell debris and precipitates are then removed by cryogenic centrifugation (3000 × g, 10 min, 4°C). Finally, the supernatant is transferred to cryogenic cryopreservation tubes and stored in a -80°C freezer or in liquid nitrogen. Theoretically, pretreatment of bile before storage is necessary because most bile specimens are relatively viscous and contain many impurities. Therefore, centrifugation of the samples before storage conveniently facilitates the extraction and identification of the exosomes in later stages. Bile extraction is a moderately traumatic operation that requires close attention to the physical condition of the patient. The whole experimental study must conform to the appropriate standards and ethical guidelines.

### 3.2. Isolation of Human Bile Exosomes

**Figure 2:**
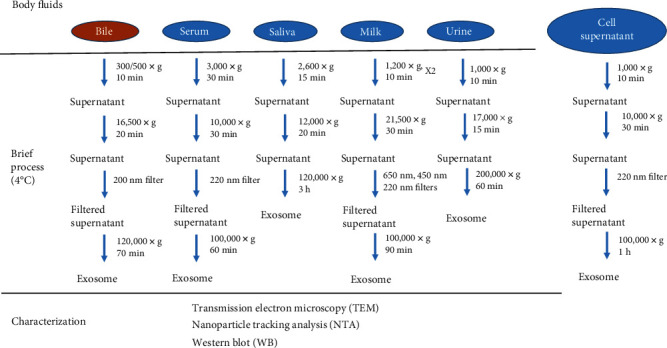
Isolation and characterization of exosomes from various humoral sources. There is no “gold-standard” protocol for exosome isolation. Exosomes from various body fluids are extracted based on a similar process: cells and cell fragments are removed by low-speed centrifugation, and the supernatant is filtered by nanofiltration; the exosomes are then precipitated by ultracentrifugation. Finally, the exosomes are washed (not shown in the figure). All operations are performed at low temperature (4°C). Exosomes are identified by three methods: TEM, NTA, and WB. It is important to note that exosomes from each humoral source have their own unique features that need to be monitored during the isolation.

Due to the lack of current exosome extraction standards, the process varies depending on body fluids (Figure 2). For example, in the case of isolation of salivary exosomes, the low viscosity of the saliva prevents it from passing through the membrane [20], and filter membranes are not used to extract urinary exosomes [21]. Compared to exosomes in other body fluids, milk exosomes require repeated extraction during the first step, which may be related to the globular fat layer in the milk [22]. However, the characteristics of exosomes differ in different reports, and the experimental conditions should be determined according to the natural conditions and sample characteristics. Despite certain specifics, the principles of exosome extraction from various body fluids are similar. In summary, low-speed centrifugation is used to remove cell debris and impurities; then, the substances that differ in diameter from the exosomes are removed by filtration through a membrane. Then, the exosomes are pelleted by ultrafast centrifugation. Finally, the exosomes enriched in the pellet can be washed with PBS. Specific samples may require pretreatment. For example, bile samples need to be centrifuged at a low speed after collection to facilitate storage, reduce the degradation of the exosomes, and ensure that the collected material is free from microbial contamination [23].

This approach has some limitations. Ultracentrifugation requires relatively specific equipment, and the process is operationally complex and requires professional training. Limited capacity of the centrifuge rotors enables processing of only a small number of samples at one time. However, the exosomes obtained by this continuous centrifugation and filtration method are undoubtedly of high purity.

Certain kits, such as ExoQuick (produced by System Biosciences), separate poorly soluble exosomes from the solution by precipitation and enable their collection by low-speed centrifugation or filtration. HansaBioMed (http://www.hansabiomed.eu/) and Life Technologies (http://www.lifetechnologies.com/exosomes) offer an array of products featuring antibodies against CD63, CD81, and CD9 for exosome capture and characterization. Bioo Scientific introduced a kit called ExoMir that uses several microfilters to filter out cellular debris based on the size of the exosomes, and exosomes larger than 30 nm in diameter are captured. The limitation of this method is the inability to purify exosomes because other substances with a similar diameter are also present in body fluids.

The introduction of some commercial kits has reduced the requirement for special instruments, which are needed for ultracentrifugation, thus reducing the requirements for the experiments and facilitating the extraction of biliary exosomes. However, different kits are based on different principles resulting in variable yield and purity of the exosomes obtained by these methods. Appropriate kits should be selected according to requirements of the study.

### 3.3. Characterization of Human Biliary Exosomes

Exosomes can be directly visualized by transmission electron microscopy (TEM) due to their structural characteristics, i.e., 30-150 nm spherical structures, and the diameter of the exosomes in the images corresponds to exosomal characteristics, thus enabling their preliminarily identification [24]. Additionally, nanoparticle tracking analysis (NTA) has been recognized as one of the means for exosome characterization in the field of exosome research. NTA is based on tracking and analysis of the Brownian motion of individual particles and calculations of the hydrodynamic diameter and concentration of the nanoparticles based on the Stokes-Einstein equation. These NTA principles provide information only on particle size distribution and particle concentration and cannot be used to distinguish exosomes from other particles. Therefore, exosome-specific markers are fluorescently labelled and are used to determine the content of specific subgroups of exosomes. Moreover, exosomes originate from intracellular sources and contain many membrane fusion and transport proteins (GTPases), tetraspanins (e.g., CD9, CD63, and CD81), and proteins involved in the biogenesis of multivesicular bodies (Alix and TSG101) [25–27]. The results obtained by Western blotting (WB) contribute to the identification of exosomes based on these specific proteins.

## 4. Pathophysiological Mechanism of Biliary Exosomes

**Figure 3:**
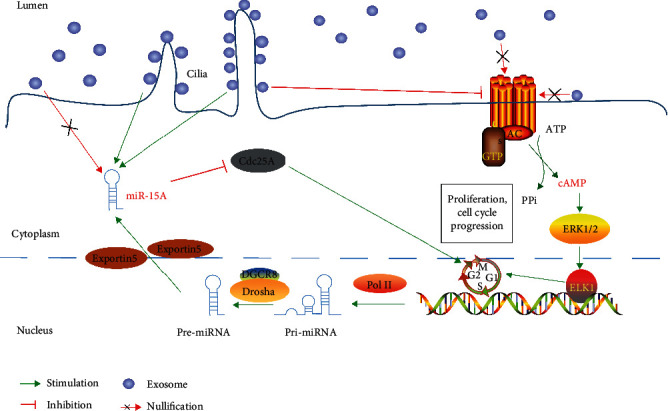
Mechanism of action of bile-derived exosomes in the normal body. Normal hepatocytes or bile duct cells secrete exosomes into bile. Exosomes in bile are enriched in the primary cilia; on the one hand, exosomes can inhibit the formation of cAMP and thus suppress the phosphorylation of ERK1/2 and ELK-1 to finally reduce the proliferation of bile duct cells. On the other hand, exosomes in contact with the cilia inhibit Cdc25A and bile duct cell proliferation by increasing miR-15A levels. All physiological processes are based on the premise that exosomes are enriched in the primary cilia.

**Figure 4:**
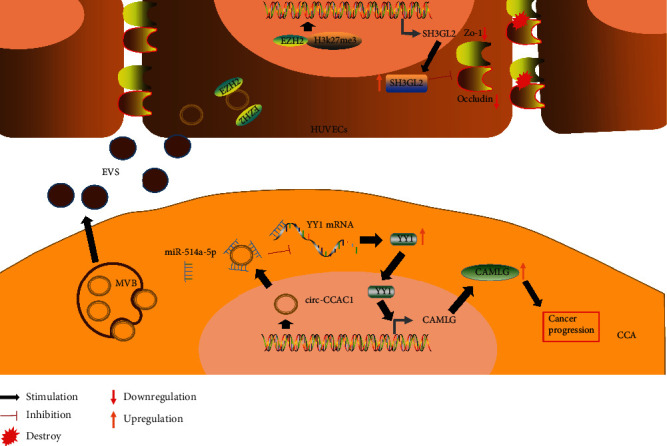
The mechanism of action of biliary exosomes in cholangiocarcinoma. circ-CCAC1, which is highly expressed in bile exosomes of patients with cholangiocarcinoma, can enhance the transcription of CAMLG in cholangiocarcinoma cells by sponging miR-514a-5p and upregulating YY1; CAMLG promotes the progression of tumour cells. circ-CCAC1 can be loaded into the exosomes and uptaken by vascular endothelial cells. This circRNA enters the cytoplasm and binds to EZH2 to reduce its intranuclear transport, decrease the formation of H3K27me3, and promote the transcription of SH3GL2; then, SH3GL2 level is increased to negatively regulate ZO-1/occludin and eventually destroy endothelial barrier function, promoting tumour angiogenesis.

A previous study confirmed that biliary exosomes attached to the primary cilia in the bile duct cause a decrease in the ratio of phosphorylated ERK1/2 to total ERK1/2, an increase in the expression of miR-15A, and a decrease in the proliferation of bile duct cells [28] (Figure 3); the study was performed using healthy animal models and demonstrated that enrichment of exosomes in the primary cilia is required for ciliary function; however, details of exosomal substances and mechanisms of action of the exosomes were not thoroughly discussed. The downstream target of miR-15A was not verified, and a tumour model was not established. We can only infer that exosomes secreted by the tumour cells in bile activated the ERK pathway based on the previously demonstrated role of the ERK pathway in the tumours, which involves promotion of cell proliferation [29]. However, because of differences in tumour types and heterogeneity of the exosomes, additional experiments are needed to confirm this hypothesis. A recent study reported that circ-CCAC1, which is expressed at a high level in biliary exosomes of patients with cholangiocarcinoma, can enhance the transcription of CAMLG in cholangiocarcinoma cells by absorbing miR-514a-5p and upregulating YY1, and CAMLG promotes the progression of the tumour cells. circ-CCAC1 can be incorporated into the exosomes and subsequently is ingested by vascular endothelial cells. This circRNA enters the cytoplasm of the cells and binds to EZH2 to reduce its intranuclear transport, decrease the formation of H3K27me3, and promote the transcription of SH3GL2 to increase the level of SH3GL2, which can negatively regulate ZO-1/occludin; these events eventually destroy the endothelial barrier function and promote tumour angiogenesis [30](Figure 4). Rafal et al. [31] have confirmed that bile duct ligation (BDL) increased release of Hh-containing exosome-enriched microparticles into plasma and bile, and all microparticles induced similar Hh-dependent changes in SEC gene expression, suggesting a novel mechanism for cirrhotic vasculopathy. Moreover, animal experiments have shown that biliary exosomes can promote the proliferation of CD4 and CD8 T cells and monocytes in the liver and can inhibit avian leucosis virus subgroup J (ALV-J) [32]. The study included only in vitro cell experiments without in vivo verification and did not further explore the mechanism of the effect of biliary exosomes on the immune function. However, the results provide an interesting reference with regard to the investigations of the relationships between biliary exosomes and immunity. Nagashima et al. [33] have also confirmed that exosomes isolated from HEV-infected cells contained detectable levels of viral ORF2 and ORF3 proteins, and the capsids of HEV particles were also individually covered by lipid membranes that resemble the lipid membranes of exosomes, which all help to elucidate the entry mechanisms and receptors for HEV infection in the future.

Most studies focused on the cargoes carried by the exosomes and specifically concentrated on noncoding RNAs. We have reviewed recent publications on the mechanism of exosome delivery in all types of humoral sources, and the findings are presented in detail in Table 1. In summary, exosomes might follow some relatively classical pathways, such as the ERK and/or FAK/Src pathways, while the main protagonists, noncoding RNAs, may mediate tumour proliferation, angiogenesis, tumour metastasis, and tumour cell apoptosis. The majority of the recent studies have been primarily interested in miRNAs and lncRNAs. The main mechanism of action of exosome-derived miRNAs includes miRNA recognition of the binding sites on the 3′-UTR of mRNAs of the target genes according to the seed sequences (5′-terminal 2-8 nucleotides) carrying RISCs. Transcriptional repression and mRNA cleavage or degradation eventually inhibit downstream gene expression or weaken or eliminate downstream gene function, resulting in indirect regulation of physiological and pathological states [34]. The secondary structure of exosome-derived lncRNAs results in protein binding, which can cause chromatin remodelling and influence the functions of transcription factors. Moreover, lncRNAs can bind to linear miRNA to indirectly influence mRNA expression or directly bind to mRNA to influence mRNA translation, fragmentation, and degradation [35, 36].

Similar to circulating noncoding RNA, the function of the whole system becomes complex due to the external structure of the exosomes and other substances present in the exosomes, such as DNA and proteins. For example, does the structure of external vesicles enhance their targeting ability? Is there any protection against the degradation of internal noncoding RNA? Interestingly, tetraspanins on the exosome surface have been reported to play an important role in the targeting of exosomes [37–39]. This family of proteins may contribute to precisely targeted therapies. Moreover, some studies have confirmed that noncoding RNAs wrapped by exosomes do not degrade after incubation at room temperature for 24 h or longer [18]. This advantage gives exosomes greater potential for use as tumour biomarkers when compared with circulating noncoding RNAs. Moreover, RNA-digesting enzymes cannot degrade RNAs carried by the exosomes with intact membrane [40]. Conversely, does the combination of membranous structures influence the binding of noncoding RNAs to target genes? Most exosomes ingested by the cells have been shown to be targeted to lysosomes, where the exosomes are subsequently degraded via biological processes [41, 42]. Does the destination of the exosomes influence the function of their internal cargoes? Additional studies are needed to answer these questions. In general, the biogenesis of exosomes is complex, varies depending on the content and cell type, and may be influenced by signalling and pathological stimuli received by the cells. Moreover, studies on exosome production and secretion should consider factors and activities that influence the mechanism and kinetics of early exosome sorting [43–45]. Currently, there are no specific reports describing the mechanism of exosome sorting in the liver or bile duct, and the applicability of general sorting rules to these structures requires confirmation. Additionally, some studies have reported that in cell culture, exosomes that are not fully released and/or are captured by the same cell require specific attention [46, 47]. Biliary exosomes may be influenced by similar fluctuating factors. Furthermore, the mechanisms governing the uptake may vary from source to source, and the mechanisms of the release of the contents of internalized exosomes are incompletely understood [48].

Because of the diversity of exosome components, the effects of noncoding RNAs may be mediated by internal proteins, lipids, or DNA; the complexity of the whole process suggests that noncoding RNAs originating from exosomes may interfere with or promote any part of the process. Thus, studies of the mechanisms of action of biliary exosomes require reasonable extrapolation based on the exosomes characterized in other sources and complete description of the related biological processes, including secretion, transport, and uptake of the exosomes, and functions that are influenced by considerable heterogeneity of the exosomes [49, 50]. Studies on biliary exosomes are limited; the mechanisms associated with biliary exosomes may be similar to those of exosomes originating from other humoral sources, and these similarities have to be gradually validated in future studies. Importantly, unique mechanisms associated with biliary exosomes may be used to facilitate the studies of biliary diseases and identify accurate treatments.

## 5. Potential Applications of Biliary Exosomes

### 5.1. Potential Biomarkers of Diseases

Bile is the most directly accessible internal environment for bile duct-related diseases. There is no doubt that all types of cytokines and extracellular vesicles may be enriched in bile regardless of bile duct-related tumours or inflammation levels, providing a theoretical basis for the studies of biomarkers. The presence of exosomes in bile has been confirmed, and these exosomes contain abundant microRNA species that are stable in bile. Thus, a diagnostic panel was developed based on bile duct carcinoma-associated microRNAs. The ROC curve confirmed a panel sensitivity of 67% and a specificity of 96% [18]. Interestingly, five miRNAs were included in this study, and a mathematical model was used to simulate a panel of five miRNAs for diagnosis, which improved the diagnostic sensitivity. However, too many molecules blindly included in a panel will reduce its specificity. Furthermore, the lncRNA species in biliary exosomes were sequenced and subjected to the GO, KEGG pathway, coexpression, receiver operating characteristic curve, and survival analyses, demonstrating that the levels of ENST00000588480.1 and ENST00517758.1 lncRNAs were significantly increased in the cholangiocarcinoma (CCA) group compared with those in the control group (benign obstruction). A combined diagnosis based on these two lncRNAs substantially improved the sensitivity and specificity of the diagnostic panel. Other studies demonstrated that these two molecules are associated with tumour staging and prognosis. In addition, a series of biogenic analyses and predictions was performed, and the p53 signalling pathway was found to be the most significantly different in the cells and associated with these lncRNAs, providing a reference for future mechanistic studies and targeted therapy. Thus, lncRNAs from exosomes may be used as potential biomarkers and therapeutic targets [19]. In addition, a comparison of biliary exosome concentrations in the case of benign and malignant biliary tract obstruction enabled researchers to define a concentration threshold, which served as the basis for discrimination between benign and malignant biliary tract obstruction. Surprisingly, the study compared the diagnostic accuracy of serum exosomes and biliary exosomes, and the accuracy of biliary exosomes was shown to be substantially higher than that of the serum exosomes (100% vs. 63.3%). Similarly, the levels of biliary exosomal circ-CCAC1, serum exosomal circ-CCAC1, and serum CA-199 (AUC = 0.857, 0.759, and 0.757, respectively) were compared, and the results showed that biliary exosomes are preferred for the diagnosis of cholangiocarcinoma, and the diagnostic effect was enhanced if biliary exosomes were combined with serum CA-199 [30]. These results suggest that biliary exosomes have good clinical application potential.

A number of recent studies have verified potential applications of the exosomes from various sources as biomarkers (Table 2). Analysis of the data indicated that biliary exosomes and exosomes from other body fluids have common stability factors and similar abilities for the transport of relatively specific substances, such as RNA, DNA, and proteins. The membrane of the vesicle-based structure of the exosomes provides better protection for the substances that otherwise are directly exposed to body fluids, and this protection may reduce the degradation of the effector substances in complex humoral environments and maintain their levels at effective and detectable doses. Moreover, some studies confirmed that tumour-derived exosomes are involved in the formation of the microenvironment before tumour metastasis and may thus serve as early warning signals of tumour metastasis [51]. Furthermore, exosomes mediate intercellular communication and carry a large amount of information about tumours; thus, exosomes may be relatively more sensitive to the progress of the tumours and to prognosis after treatment. Apparently, biliary exosomes are more accurate predictors of biliary tract-related diseases than other humoral exosomes. Furthermore, bile is the most directly accessible internal environment for these diseases, especially for CCA. Therefore, exosomes secreted by tumour cells are expected to be enriched in bile, suggesting their use for early diagnosis. Moreover, the relatively closed internal environment of the bile duct reduces the effect of other interfering factors. Hence, additional studies are needed to confirm the effectiveness and specificity of the exosomes as biomarkers that can be included in the development of new panels for diagnosis and prognosis of various diseases.

However, these conclusions cannot be blindly generalized or extended. A recent study on the diagnosis of pancreatic ductal adenocarcinoma (PDAC) confirmed that a higher frequency of mutant KRAS was detected in the circulating exosome-derived DNA in PDAC patients and in the vast majority of healthy subjects [52]. This result suggests that generalization of the findings obtained using exosome-derived molecules requires scrutiny and validation by a rigorous experimental process.

### 5.2. Potential Therapeutic Applications for Diseases

Exosomes are membranous vesicles with a diameter of 30-150 nm secreted by cells. Exosomes play important roles in cell-to-cell communication through the biological processes involved in their generation and secretion and targeting of receptor cells [53, 54]. The structure and functions of exosomes suggest that they have great therapeutic potential [55]. For example, intracellular exosome generation and extracellular exosome release enable exosomes to serve as carriers of intracellular substances and as specific biomarkers that reflect the state of the cells or tissues. The characteristics of targeting receptor cells enable precise drug delivery via exosomes to the target cells or tissues, which may increase the concentration of the drugs in the target tissues and thus reduce the toxicity and side effects of the drugs in other tissues. Furthermore, the outer membrane of these vesicles may increase the stability of the therapeutic system, preventing drug degradation and irrelevant deposition [56]. Recent studies on therapeutic exosomes have been divided into two categories: natural exosomes produced within an organism and externally modified exosomes obtained from conditioned medium in specific cultures or special external treatments (drugs or gene modification) [55]. Although the mechanism of exosome therapy is not well understood, its potential is gradually being demonstrated.

Exosomes with certain characteristics can be ideal therapeutic tools. However, certain limitations have been demonstrated. For example, it is difficult to track the exosome transport process and distribution in vivo. Fortunately, some researchers have developed a means of direct labelling of the exosomes through gas-coated gold nanoparticles (GNPs) and identified the distribution of these exosomes using computed tomography to determine the best drug delivery routes [57]. Another limitation to the therapeutic effect of exosomes is due to unidentified substances in body fluids that can invade and affect exosomes. Interestingly, this limitation can be overcome by enveloping exosomes with nanofilms composed of supramolecular complexes of ferric ions (Fe^3+^) and tannic acid. Additionally, nanofilms enable the retention of the inherent size and chemical composition of the exosomes thereby promoting the controlled release of drugs [58]. The clinical application of exosomes faces the challenge of insufficient targeting, and a research team has developed a dual-function exosome-based superparamagnetic nanoparticle cluster as a delivery vehicle for targeting drugs for cancer therapy; this vehicle manifested greater responsiveness than that of a single superparamagnetic nanoparticle, thus enhancing the targeting ability of the tumours [59]. The application of the exosomes in clinical treatment is expected to encounter a series of problems, including complex operation due to high difficulty and high cost, which will require further studies to resolve.

The c(RGDyK) peptide was conjugated to the exosomes in a certain manner, and these exosomes were used as carriers to deliver curcumin in a targeted manner in an ischaemic stroke model in mice; this treatment produced anti-inflammatory effects in the brain and overcame the inability of drugs to pass the blood-brain barrier [60]. Similarly, macrophage exosomes were used as natural nanocarriers to deliver proteins for the treatment of inflammatory encephalopathy [61]. The carrier must be improved during the application process based on the immediate situation to enhance transport efficiency and effect. This improvement requires the synchronous development of nanoengineering technology. Cargo-loaded exosomes have broad therapeutic prospects. For example, docosahexaenoic acid (DHA) induced miR-23b and miR-320b overexpression by changing the exosome secretion levels and reducing the expression of the angiogenesis-related target genes (PLAU, AMOTL1, NRP1, and ETS2) to ultimately inhibit tumour progression [62]. In addition, exosome therapy may be closely associated with tumour immunity. Some studies reported that phagocytosis of tumour cells by phagocytes was inhibited because of the interaction between CD47 on the tumour surface and the signal regulatory protein (SIRPalpha) on macrophages [63, 64]. The interaction between CD47 and SIRPalpha was antagonized using SIRPalpha-exosomes to promote phagocytosis of the tumour cells [65]. Moreover, CpG DNA-modified exosomes (CpG-SAV-exo) were generated and used to form an exosome-based tumour antigen-adjuvant codelivery system, which may be used in cancer immunotherapy [66]. Interestingly, exosomes may also be used to explain the therapeutic effect of exercise on metabolic diseases, such as obesity and type 2 diabetes (T2DM). For example, a study suggested that the benefits of exercise are mediated by exosomes and/or microvesicles that function in an autocrine, paracrine, and/or endocrine manner and have therapeutic effects on obesity and diabetes [67]. Various new technologies were used to generate artificial therapeutic exosomes, mimicking the structural characteristics of endogenous exosomes. These exosomes were modified according to the needs of individual patients and may have broad therapeutic significance [68].

Bile is a special fluid secreted by the liver and transported through the bile ducts. Bile does not reach the whole body; therefore, characteristics of bile can be used to accurately diagnose diseases of the biliary tract. There is no doubt that biliary exosomes may have possible therapeutic uses and effects based on principles described in the present review. Bile is the most directly accessible contact environment in the diseases of the bile duct system; thus, bile-specific exosomes may produce better therapeutic effects. For example, circ-CCAC1 in biliary exosomes may play an important role in CCA progression; therefore, suppression of circ-CCAC1 expression or blocking the transmission of exosomal circ-CCAC1 might be a novel therapeutic strategy for CCA [30]. Furthermore, bile enters the duodenum via the bile duct, providing certain therapeutic potential for intestinal diseases. Physiological circulation through the liver and intestine may also produce lasting effects. However, extraction of bile is a relatively complex and difficult process, which is, to an extent, the main limiting factor of studies on biliary exosomes.

## 6. Conclusion and Perspectives

**Figure 5:**
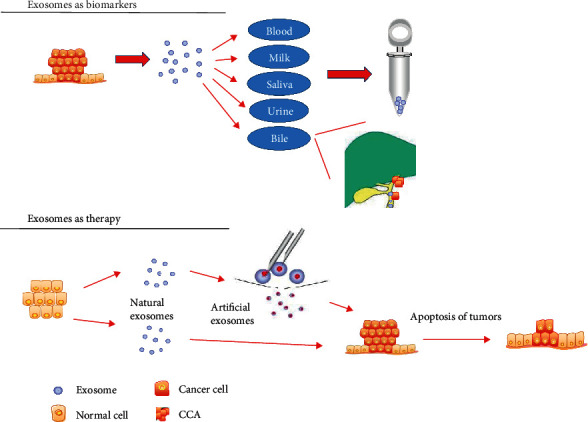
Potential clinical application of exosomes. Exosomes are produced by living cells, carry a variety of active molecules, and are ubiquitously present in various body fluids. Exosomes may be detected in the microenvironment before tumour metastasis and reflect tumour proliferation and metastasis; therefore, they can be used as biomarkers for early diagnosis and prognosis of tumours. Because of specialized vesicle structure, exosomes are stable carriers of therapeutic drugs with increased targeting ability. Therapeutic exosomes can be divided into two categories: natural exosomes secreted directly in body fluids and artificially engineered exosomes.

In brief, we reviewed the biogenesis and development of exosomes, provided the initial theoretical basis for future mechanistic studies, summarized the isolation and identification of biliary exosomes for the first time, and highlighted the mechanisms and principles of the effects of exosomes in bile and the clinical application potential of the exosomes (Figure 5). We described nonbiliary exosomes as models and reasonably extended the results of the breakthrough studies to bile-derived exosomes. These considerations are based on the premise of a systematically elaborated mechanism and application of exosomes. Moreover, we provided references for future studies on biliary exosomes via a literature review that includes a general research strategy and theoretical basis. The studies of biliary exosomes remain limited due to the moderately traumatic acquisition of bile and numerous bile components, such as bile salts, which may influence the effects of the exosomes. Additionally, the range of bile action is relatively limited, in contrast to that of the blood, which circulates throughout the body. However, these unresolved problems do not diminish the potential value of biliary exosomes in diagnosis and treatment of biliary tract-related diseases; additional in-depth studies are needed to thoroughly investigate biliary exosomes.

## Figures and Tables

**Table 1:** Mechanism of action of exosomes from various humoral sources.

Source	Disease type	Related molecule	Signalling pathway	Function	Ref.
Bile	Normal physiology	miR-15A	ERK	Exosomes in bile interact with primary cilia to reduce the proliferation of bile duct cells through the ERK pathway and miR-15A.	[28]
	Normal physiology	ALV-J	—	Biliary exosomes promote the proliferation of CD4^+^ and CD8^+^ T cells and monocytes in the liver, and can inhibit ALV-J.	[32]

Bile, cell line	Cholangiocarcinoma	circ-CCAC1	circ-CCAC1/miR-514a-5p/YY1/CAMLG axis EZH2/SH3GL2/ZO-1/occludin	The circ-CCAC1-carrying EVs released by cholangiocarcinoma cells were absorbed by endothelial cells. circ-CCAC1 was able to destroy vascular barrier and angiogenesis via the regulation of EZH2/SH3GL2/ZO-1/occludin.	[30]

Serum, cell line	Prostate cancer	PKM2, CXCL12	HIF-1*α*	Exosome-mediated PKM2 upregulated BMSC CXCL12 by HIF-1*α*-dependent fashion to complete bone metastasis of prostate cancer.	[69]
	Liver fibrosis	lncRNA-H19	—	Exosomal lncRNA-H19 promotes cholestatic liver fibrosis by promoting the differentiation and activation of HSCs.	[70]
	Cholestatic liver injury	lncRNA-H19	ERK1/2, AMPK, SHP	Uptake of H19-carrying exosomes from cholangiocytes suppresses SHP expression by inhibiting promotor activity and destabilizing SHP mRNA in hepatocytes. Downregulation of SHP expression results in increase of bile acid synthesis and eventually causes cholestatic liver injury.	[71]
	Bladder tumour	lncRNA-UCA1	EMT	Hypoxic exosomal lncRNA-UCA1 promoted tumour growth and progression in vitro and in vivo through EMT.	[72]

Tissue, cell line	Hepatocellular carcinoma	LOXL4	FAK/Src pathway	Exosome-mediated secretion of LOXL4 by modulating the FAK/Src pathways and angiogenesis in HCC, plays the role of tumour metastasis.	[73]
	Gastric cancer	HMGB1/TLR4	NF-*κ*B pathway	Gastric cancer-derived exosomes carry high mobility group box-1 (HMGB1), which interacts with Toll-like receptor 4 (TLR4) to activate NF-*κ*B and induce neutrophil autophagy, thereby promoting gastric cancer cell migration.	[74]
	Inflammatory bowel disease	ANXA1	FPR1 and FPR2/ALX	Endogenous annexin A1 (ANXA1) is released as a component of intestinal epithelial EVs that activate wound repair circuits.	[75]

Cell line	Clear cell renal cell carcinoma	miR-19b-3p, PTEN, CD103, E-cadherin, N-cadherin, vimentin, twist	EMT	CSC exosomes transport miR-19b-3p to CCRCC cells and initiate EMT to promote metastasis. CD103 enables tumour to target lung.	[76]
	Osteochondral defects	CD73, IL-1*β*, TNF-*α*	AKT, ERK	MSC exosomes achieve osteochondral regeneration through coordinated mobilization of multiple cell types and activation of multiple cellular processes.	[77]

Urine	Diabetes	DMBT1	/	USC-exos may promote angiogenesis by transferring DMBT1 protein.	[78]

Breast	Breast cancer	TGF*β*2, E-cadherin, alpha-smooth muscle actin (*α*-SMA), filamentous- (F-) actin, vimentin	EMT	Breast exosomes with high TGF*β*2 expression can induce changes in benign and malignant breast epithelial cells.	[79]

**Table 2:** Biomarkers in exosomes from various humoral sources.

System	Diseases	Source	Molecule	Application	AUC value	Ref.
Digestive system	Malignant biliary stenosis	Bile	EV concentrations	Diagnosis	1.0	[17]
	Cholangiocarcinoma	Bile	Multi-miR markers	Diagnosis	—	[18]
	Cholangiocarcinoma	Bile	ENST00000588480.1, ENST00000517758.1	Diagnosis Prognosis	0.709	[19]
	Cholangiocarcinoma	Bile	Exosomal circ-CCAC1	Diagnosis	0.857	[30]
	Liver cancer	Plasma	Four exosomal tsRNAs	Diagnosis	—	[80]
	Pancreas cancer	Serum tissue	GPC1(+) crExos	Diagnosis	1.0	[81]
	Pancreas cancer	Blood	Glypican-1, CD63	Diagnosis	0.989	[82]
	Gastric cancer	Plasma culture media	Exosomal lncUEGC1	Diagnosis	0.876	[83]

Urogenital system	High-grade prostate cancer	Urine	ExoDx Prostate IntelliScore	Diagnosis	0.70	[84]
	Prostate cancer	Urine	miR-196a-5p, miR-501-3p	Diagnosis	0.73, 0.69	[85]
	High-grade prostate cancer	Urine	ExoDx Prostate IntelliScore	Diagnosis	0.77	[86]
	Prostate cancer	Urine	Exosomal metabolites	Diagnosis Prognosis	—	[87]
	Castration-resistant prostate cancer	Plasma	miR-1290, miR-375	Prognosis	0.68	[88]
	Bladder cancer	Urine	MALAT1,PCAT-1, SPRY4-IT1	Diagnosis Prognosis	0.813	[89]
	Cervical cancer	Plasma	Let-7d-3p, miR-30d-5p	Diagnosis	0.828	[90]
	Breast cancer	Serum	miR-21, miR-222, miR-200c	Diagnosis	—	[91]

Respiratory system	Lung cancer	Plasma	CD151, CD171, TSPAN8	Diagnosis	0.68, 0.60, 0.60	[92]
	Asthma	Culture medium	EPO, MBP, ECP	Diagnosis	—	[16]

Nervous system	Alzheimer's disease	Blood CSF	Abeta42, T-tau, P-T181-tau	Diagnosis	0.98	[93]
	Relapsing remitting multiple sclerosis	CSF	Exosomal proteins	Diagnosis	—	[94]

Other diseases	Melanoma	Plasma	Melanoma-derived exosomes	Diagnosis Prognosis	—	[95]
	Pheochromocytoma Paraganglioma	Serum	Exosomal dsDNA	Diagnosis	—	[96]
	Down syndrome	Blood	Abeta1-42, P-T181-tau, P-S396-tau	Diagnosis	—	[97]
	Preeclampsia	Plasma	PlGF, CTB-TIMP1, AV-PAI1	Diagnosis	0.96	[98]
	Multiple myeloma	Serum	Let-7b, miR-18a	Diagnosis prognosis	—	[99]

## Data Availability

The data used to support the findings of this study are available from the corresponding author upon request.

## Abbreviations

MVBs: Multivesicular bodies
EVs: Extracellular vesicles
miRNAs: MicroRNA
lncRNA: Long noncoding RNA
ERCP: Endoscopic retrograde cholangiopancreatography
IR: Interventional radiology
PBS: Phosphate-buffered saline
TEM: Transmission electron microscopy
NTA: Nanoparticle tracking analysis
WB: Western blotting
ALV-J: Avian leucosis virus subgroup
RISC: RNA-induced silencing complex
CCA: Cholangiocarcinoma
PDAC: Pancreatic ductal adenocarcinoma
KRAS: Kirsten rat sarcoma viral oncogene
GNPs: Gold nanoparticles
DHA: Docosahexaenoic acid
SIRPalpha: Signal regulatory protein
CpG-SAV-exo: CpG DNA-modified exosome
T2DM: Type 2 diabetes
PKM2: Pyruvate kinase isozyme type M2
BMSC: Bone marrow stromal cell
HSC: Hepatic stellate cell
EMT: Epithelial-mesenchymal transition
LOXL4: Lysyl oxidase like protein 4
HCC: Hepatocellular carcinoma
HMGB1: High mobility group box-1
TLR4: Toll-like receptor 4
ANXA1: Annexin A1
FPR: Formyl peptide receptor
PTEN: Gene of phosphate and tension homology deleted on chromosome ten
CSC: Cancer stem cell
CCRCC: Clear cell renal cell carcinoma
TNF-*α*: Tumour necrosis factor alpha
TGF*β*2: Transforming growth factor beta 2
*α*-SMA: Alpha-smooth muscle actin
MSC: Mesenchymal stem cell
IL-1*β*: Interleukin-1*β*
DMBT1: Deleted in malignant brain tumours
AUC: Area under curve.
